# Gastric Microbiota from Populations with High Risk of Gastric Cancer in Nariño, Colombia

**DOI:** 10.3390/ijms27188346

**Published:** 2026-09-19

**Authors:** Siomy Portilla, Alvaro Pazos, Vanessa Pabón, Kevin Andrés Guzmán

**Affiliations:** 1 Department of Biology, Faculty of Exact and Natural Sciences, University of Nariño, Pasto 520002, Colombia; siomyp14@gmail.com (S.P.); alpazmo@udenar.edu.co (A.P.); 2 Public Health Research Group, Centro de Estudios en Salud(CESUN), University of Nariño, Pasto 520002, Colombia; lvpabonf@sena.edu.co; 3 Tecnoacademia de Tuquerres, SENA Regional Nariño, Tuquerres 525520, Colombia

**Keywords:** *Helicobacter pylori*, gastric microbiota, stomach neoplasms, *16S* rRNA gene, Oxford Nanopore sequencing

## Abstract

Gastric cancer is a major cause of cancer mortality in Colombia, particularly in the Andean region of Nariño. *Helicobacter pylori* is the main etiological factor, but its oncogenic potential depends on bacterial variability, host susceptibility, and interactions with the gastric microbiota. This study evaluated the composition and diversity of the gastric microbiota in patients with preneoplastic lesions. Gastric biopsies from 30 patients (24 with non-atrophic gastritis (NAG); 6 with atrophic gastritis and intestinal metaplasia (AGIM)) were analyzed by full-length *16S* rRNA gene sequencing using Oxford Nanopore MinION. Taxonomic classification was performed with Kraken and SILVA v132. Alpha and beta diversity were analyzed in R, and differential abundance was assessed using ANCOM-BC2 and DESeq2. Sequencing generated 532,734 high-quality reads, from which 912 genera across 46 phyla were identified. *Proteobacteria*, *Campylobacterota*, and *Actinobacteriota* predominated, with *Helicobacter*, *Pseudomonas*, and *Cutibacterium* as the most abundant genera. *Helicobacter* abundance, rather than lesion type, significantly influenced community structure. High *Helicobacter* abundance was associated with greater alpha diversity and distinct beta diversity. No significant differences were observed between NAG and AGIM. *Prevotella* and *Alteromonas* were enriched in AGIM, whereas *Pseudomonas* increased approximately 64-fold in samples with low *Helicobacter* abundance. These findings indicate that *Helicobacter* abundance was significantly associated with differences in gastric microbial community structure, suggesting that microbial interactions may contribute to early gastric carcinogenesis.

## 1. Introduction

Gastric cancer (GC) represents one of the foremost challenges in global public health, with approximately 1.09 million new cases and 769,000 deaths annually, making it the fifth most common cancer and the fourth leading cause of cancer-related mortality [1,2]. In Colombia, this neoplasm ranks first in cancer mortality and displays a markedly heterogeneous geographic distribution [3]. In the department of Nariño, the incidence is particularly high in Andean zones, with rates ranging from 40 to 150 cases per 100,000 inhabitants, compared with only 6 on the Pacific coast [4,5]. This pattern, known as the “Colombian enigma,” has established Nariño as a key epidemiological model for studying GC etiology [6,7].

GC etiology involves a complex interplay of genetic, environmental, and particularly infectious factors. *Helicobacter pylori* (*H. pylori*) is the principal etiological agent and has been classified as a Group I carcinogen by the International Agency for Research on Cancer [3,8,9]. This bacterium has coevolved with humans since their migration out of Africa approximately 60,000 years ago, establishing chronic colonization of the gastric mucosa [10]. Infection can trigger a pathological sequence known as the Correa cascade, which encompasses chronic gastritis, gastric atrophy, intestinal metaplasia, and dysplasia, stages that may culminate in gastric adenocarcinoma [3]. However, despite *H. pylori* infection being the most prevalent bacterial infection worldwide and colonizing more than 50% of the global population, only 1–3% of infected individuals develop GC, indicating that additional factors contribute to individual susceptibility, including host genetic characteristics, environmental and dietary exposures, and the composition of the accompanying gastric microbiota [11,12,13,14,15].

Recent advances in genetic sequencing, particularly of the *16S* ribosomal RNA gene, along with metagenomic tools, have transformed the study of the microbiota [16]. These technologies enable more precise identification of microorganisms present in a given niche, determination of their relative abundance, and assessment of their functional potential, thereby improving disease diagnosis, advancing personalized medicine, and enabling evaluation of predisposition to conditions such as GC [17,18].

Advances in the study of the gastric microbiota have revealed that microorganisms inhabiting the gastric niche alongside *H. pylori* may play a key role in gastric carcinogenesis [19]. In some cases, GC can develop even in the absence of *H. pylori*; indeed, colonization by this pathogen declines progressively in individuals with precancerous lesions and eventually disappears in advanced stages such as adenocarcinoma [20]. Nevertheless, during chronic infection, *H. pylori* alters the gastric environment, promoting the growth of bacteria that would not normally thrive under acidic conditions [21]. This alteration results in dysbiosis characterized by the loss of bacteria with a potentially protective role, such as *Porphyromonas*, *Neisseria,* and *Prevotella*, and by the expansion of potentially harmful taxa, including *Clostridium*, *Bacteroides*, *Veillonella*, and *Fusobacterium* [19,20,21,22,23]. This altered microbial composition may sustain a state of chronic inflammation, generate carcinogenic compounds such as N-nitroso, and activate molecular pathways associated with tumor progression [19].

Despite recent progress, conclusive evidence establishing a clear relationship between gastric microbiota composition and GC-associated preneoplastic gastric lesions remains lacking. In particular, no characteristic microbial profile has been defined for early stages such as non-atrophic gastritis (NAG) and atrophic gastritis with intestinal metaplasia (AGIM), nor is it fully understood how the accompanying microbiota modulates *H. pylori* pathogenicity, particularly in populations with high GC incidence such as that of Nariño, Colombia. Elucidating these interactions is therefore essential to understanding the role of the microbiota in the development of precancerous gastric lesions. Accordingly, the aim of this study was to determine the composition and diversity of the gastric microbiota according to the type of gastric lesion (NAG or AGIM) in patients at high risk of gastric cancer from Nariño, Colombia.

## 2. Results

Sequencing with the MinION Mk1C device (Oxford Nanopore, Oxford, United Kingdom) generated more than 600,000 reads in total, of which 532,734 had a quality score ≥ Q15. These high-quality reads were used for subsequent taxonomic classification. The identified bacterial taxa comprised 46 phyla, with taxonomic assignments resolved to the genus level, 100 classes, 235 orders, 363 families, and 912 genera. This level of classification reflects the broad microbial diversity detected in the samples analyzed by third-generation sequencing. These results correspond to a cohort of 30 patients from whom the gastric samples were obtained. Patient sample ID, age, sex, municipality of origin, and histopathological diagnosis are presented in Table 1.

At the phylum level, the bacterial community showed the highest numbers of assigned reads for *Proteobacteria*, *Campylobacterota*, and *Actinobacteriota*, whereas other phyla such as *Firmicutes*, *Acidobacteriota*, and *Desulfobacterota* were represented by fewer reads. At the class level, *Gammaproteobacteria*, *Campylobacteria*, and *Actinobacteria* accounted for the highest read counts, consistent with the predominant phyla mentioned above. At the order level, the highest read counts were assigned to *Pseudomonadales*, *Campylobacterales*, and *Propionibacteriales*. Similarly, at the family level, the most abundant families were *Pseudomonadaceae*, *Helicobacteraceae*, and *Propionibacteriaceae*, families previously associated with the gastric environment and pathological conditions. Finally, at the genus level, a considerable number of reads were assigned to genera of clinical relevance, including *Pseudomonas*, *Helicobacter*, and *Cutibacterium*. The number of reads associated with these genera varied among patients, suggesting differences in the composition of the gastric microbiota across individuals.

The relative abundance of the most representative taxa was analyzed at different taxonomic levels. For phyla, those with a relative abundance exceeding 1% were considered, whereas for families, those exceeding 5% were included. For genera, the 20 most abundant were evaluated. These comparisons were performed according to group type and *Helicobacter* categorization.

At the phylum level, the bacterial community was dominated by *Proteobacteria*, which showed the highest relative proportion, followed by *Campylobacterota* and *Actinobacteriota*. At the class level, the predominant groups were *Gammaproteobacteria*, *Campylobacteria*, and *Actinobacteria*, while other classes were detected in reduced proportions. At the order level, *Campylobacterales* was the most abundant, followed by *Pseudomonadales* and *Propionibacteriales*. At the family level, *Helicobacteraceae* predominated, followed by *Pseudomonadaceae* and *Propionibacteriaceae*. Finally, at the genus level, the most abundant taxon was *Helicobacter*, followed by *Pseudomonas* and *Cutibacterium*.

**Figure 1:**
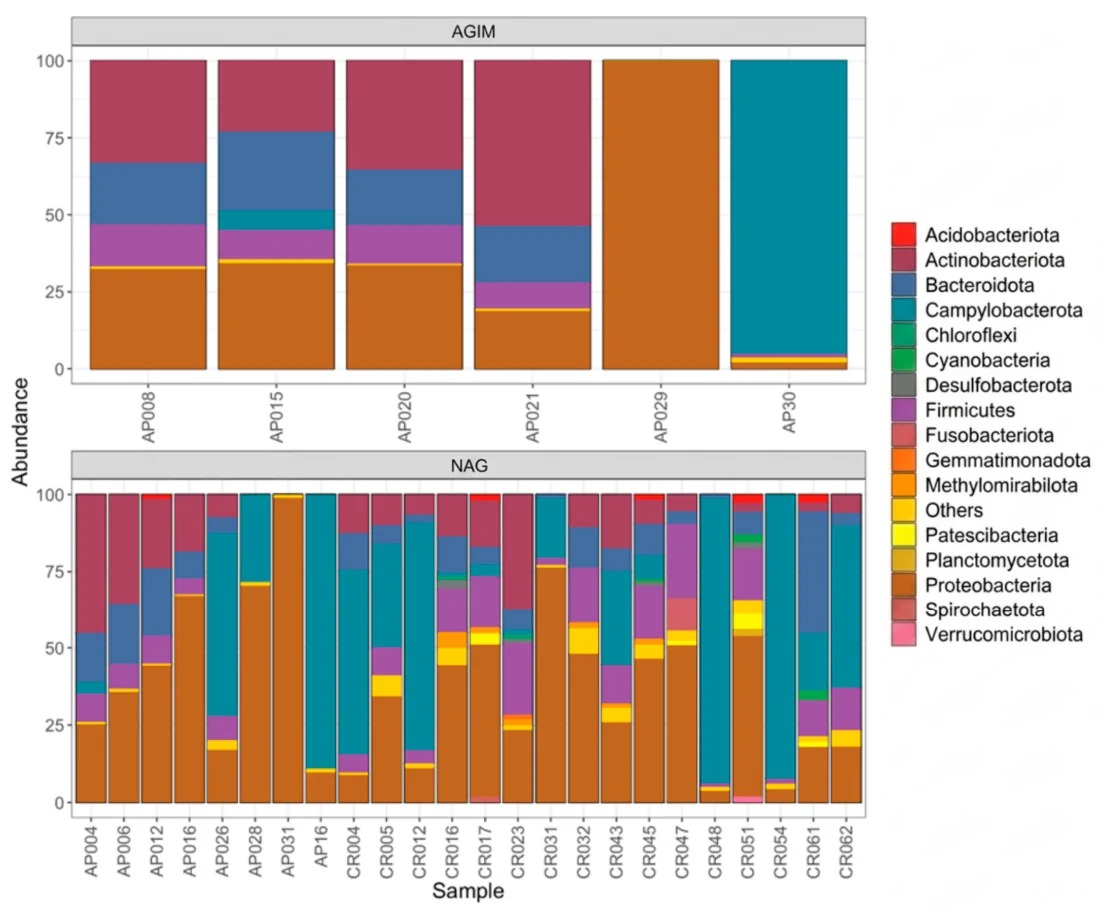
The relative abundance of bacterial phyla (>1%) by group type. Patients with atrophic gastritis with intestinal metaplasia (AGIM, **upper panel**) and non-atrophic gastritis (NAG, **lower panel**). Each bar represents an individual sample, and colors indicate the corresponding bacterial phylum.

Analysis of relative abundance at the phylum level revealed differences in the composition of the gastric microbiota between patients in the AGIM and NAG groups. In the AGIM group, fewer phyla were identified, without uniform dominance across samples. Sample AP029 was dominated almost exclusively by *Proteobacteria*, whereas AP030 showed a high proportion of *Campylobacterota*. In the remaining samples (AP008, AP015, AP020, and AP021), a more distributed composition was observed, with contributions from *Proteobacteria*, *Actinobacteriota*, and *Bacteroidota* as the principal phyla, and a lower representation of *Firmicutes*. In the NAG group, the composition was notably more heterogeneous, with a greater number of phyla detected per sample. Although *Proteobacteria* remained predominant in several samples, in other samples *Campylobacterota* reached the highest relative abundance. *Firmicutes* and *Bacteroidota* were present in variable proportions. Notably, the phyla *Acidobacteriota* and *Cyanobacteria* were detected exclusively in the NAG group, contributing to the greater phylum-level richness observed in this group compared with AGIM (Figure 1).

**Figure 2:**
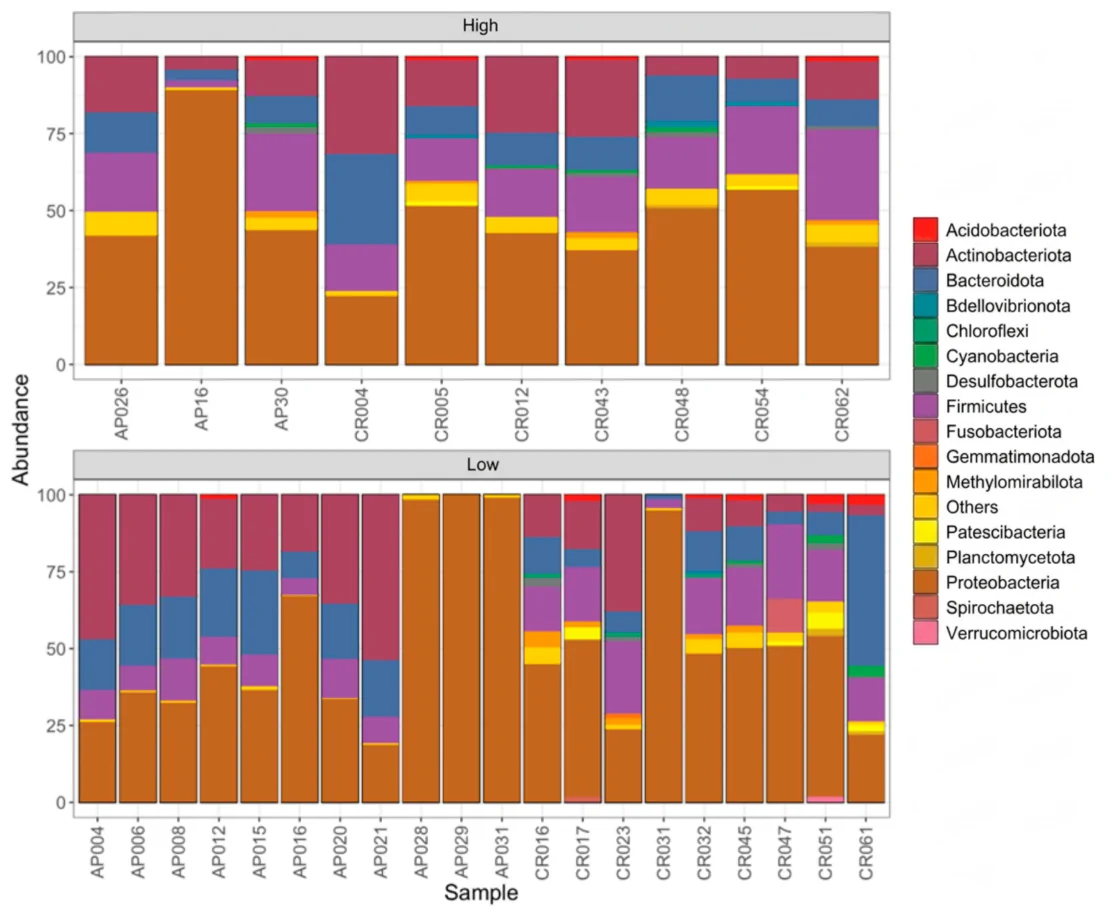
The relative abundance of bacterial phyla (>1%) according to *Helicobacter* categorization. Patients with a high *H. pylori* load (High, **upper panel**) and a low *H. pylori* load (Low, **lower panel**). Each bar represents an individual sample, and colors indicate the corresponding bacterial phylum.

In the high *Helicobacter* group, a marked dominance of *Proteobacteria* was observed in the majority of samples, accompanied by variable representation of *Firmicutes*, *Actinobacteriota*, and *Bacteroidota*. Similarly, in the low group, *Proteobacteria* was also the dominant phylum, with exclusive predominance in AP029 and near-exclusive predominance in AP028 and AP031; however, in most samples, it coexisted with considerable proportions of *Actinobacteriota*, *Bacteroidota*, and *Firmicutes*, resulting in a more even distribution among phyla (Figure 2).

**Figure 3:**
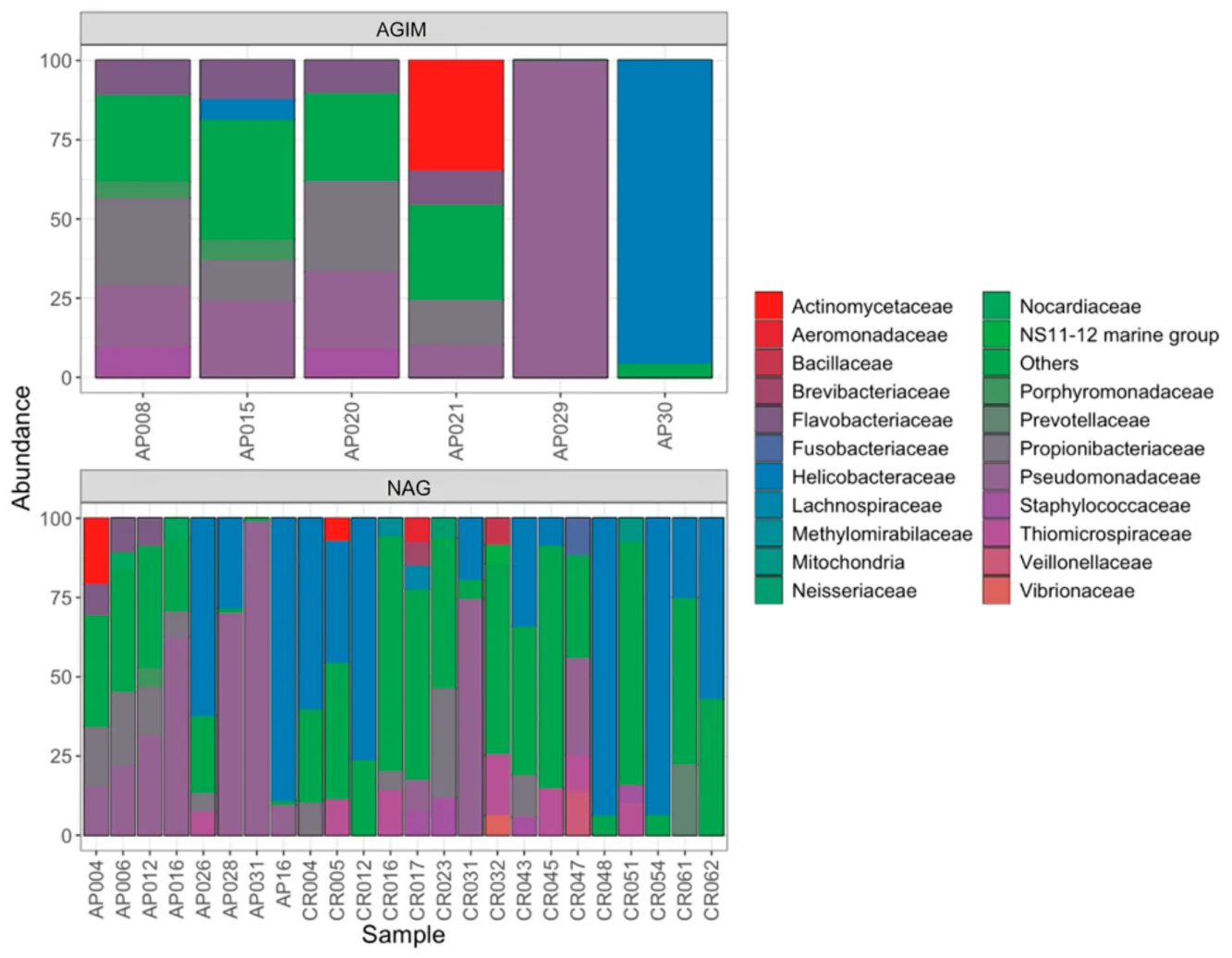
The relative abundance of bacterial families (>5%) by group type. Patients with atrophic gastritis with intestinal metaplasia (AGIM, **upper panel**) and non-atrophic gastritis (NAG, **lower panel**). Each bar represents an individual sample, and colors indicate the corresponding bacterial family.

In the AGIM group, the bacterial community showed low compositional variability, although differences were observed in the predominant families depending on the sample analyzed. In most cases, the community structure was dominated by the “others” category, accompanied by variable proportions of *Pseudomonadaceae*, *Propionibacteriaceae*, and to a lesser extent, *Flavobacteriaceae*. However, particular dominance patterns were observed in some samples: *Actinomycetaceae* predominated in AP021, *Pseudomonadaceae* in AP029, and *Helicobacteraceae* in AP030, whereas *Porphyromonadaceae* and the marine group NS11-12 were present only in low proportions. In contrast, the NAG group exhibited greater heterogeneity among bacterial profiles. A dominance of *Helicobacteriaceae* was observed, followed by *Pseudomonadaceae* and in lower proportions *Propionibacteriaceae* and *Actinomycetaceae*. Additionally, several bacterial families were exclusively detected in this group, including *Brevibacteriaceae*, *Fusobacteriaceae*, *Prevotellaceae*, *Vibrionaceae*, *Veillonellaceae*, and *Neisseriaceae*, whose presence suggests greater ecological complexity in the microbiota associated with NAG (Figure 3).

**Figure 4:**
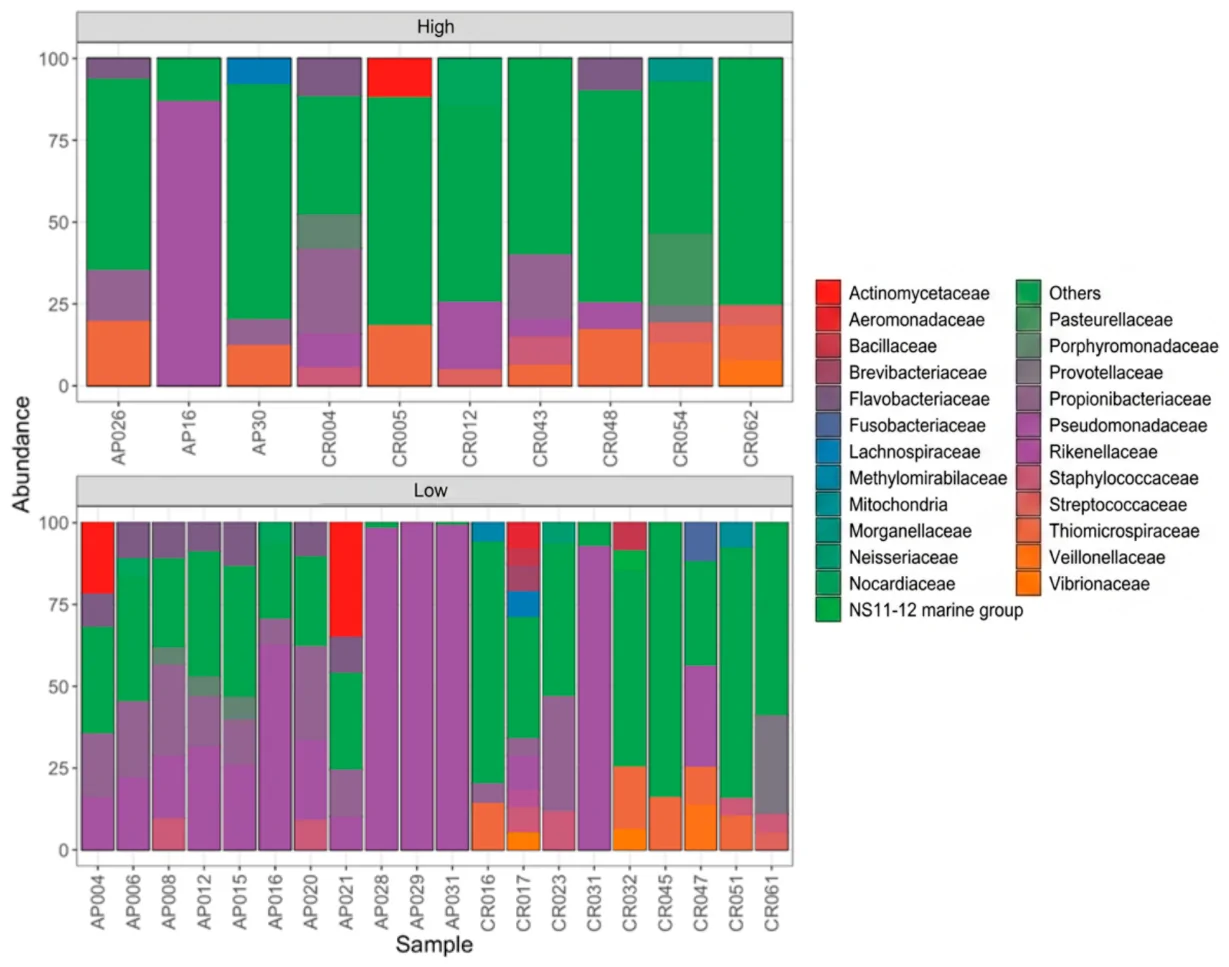
The relative abundance of bacterial families (>5%) according to *Helicobacter* categorization. Patients with a high *H. pylori* load (High, **upper panel**) and a low *H. pylori* load (Low, **lower panel**). Each bar represents an individual sample, and colors indicate the corresponding bacterial family.

In the high *Helicobacter* group, relative abundance analysis at the family level revealed a marked dominance of the “other” category in the majority of samples (>50%), indicating a high proportion of reads that could not be assigned to any of the main bacterial families identified. *Pseudomonadaceae* showed a variable contribution and was nearly dominant in sample AP16, while *Flavobacteriaceae* and *Thiomicrospiraceae* were detected in lower proportions. Likewise, certain families were observed exclusively in individual samples: *Actinomycetaceae* (CR005), *Veillonellaceae* and *Vibrionaceae* (CR062), and *Lachnospiraceae* (AP30). Sample CR054 showed a distinctive composition, with the detection of *Porphyromonadaceae*, *Prevotellaceae*, and *Pasteurellaceae*, reflecting the taxonomic heterogeneity of this group. In the group with low *Helicobacter* categorization, *Pseudomonadaceae* and the “other” category maintained broad representation, accompanied by *Propionibacteriaceae* and *Actinomycetaceae* in lower proportions. Likewise, families exclusive to individual samples were identified, including *Fusobacteriaceae* (CR047), *Mitochondria* (CR051), and *Prevotellaceae* (CR061). Additionally, this group presented families not detected in the high *Helicobacter* group, including *Methylomirabilaceae* (CR016), *Aeromonadaceae* and *Brevibacteriaceae* (CR017), and *Streptococcaceae* (CR061) (Figure 4).

**Figure 5:**
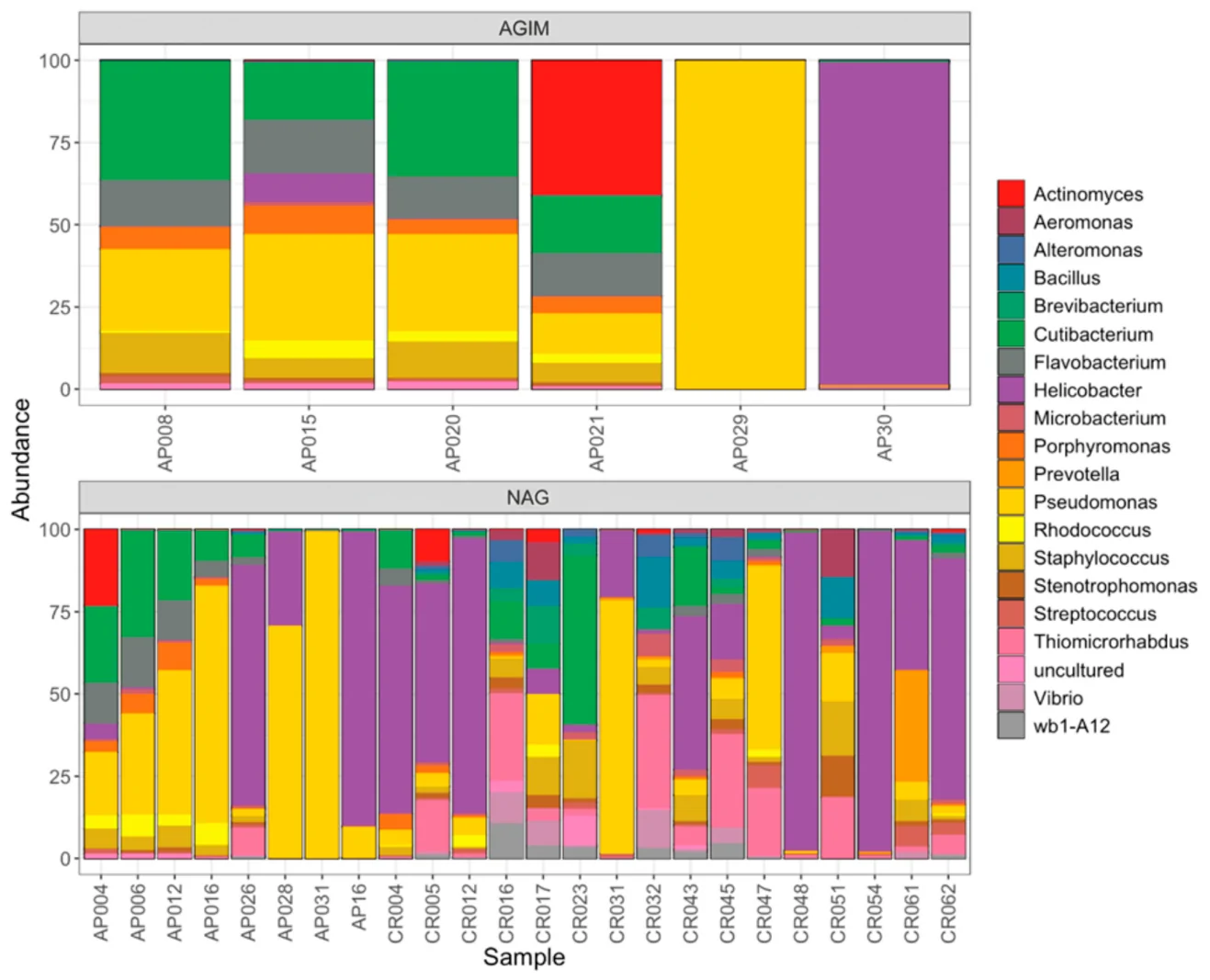
The relative abundance of the 20 most abundant bacterial genera by group type. Patients with atrophic gastritis with intestinal metaplasia (AGIM, **upper panel**) and non-atrophic gastritis (NAG, **lower panel**). Each bar represents an individual sample, and colors indicate the corresponding bacterial genus.

In the AGIM group, the gastric microbiota showed marked heterogeneity among individuals, with low diversity profiles and pronounced dominance of individual genera. *Pseudomonas* was the predominant genus in several samples, with the highest representation in AP029, while *Cutibacterium* constituted a relevant fraction in others. Genera including *Flavobacterium*, *Porphyromonas*, *Rhodococcus*, *Staphylococcus*, and *Stenotrophomonas* were identified at lower abundances. Notably, sample AP30 showed near-exclusive dominance by *Helicobacter*, reflecting intense colonization by this genus in that individual. In contrast, the NAG group exhibited microbial communities with greater genus-level richness and compositional variability among samples. *Helicobacter* was the dominant genus in several samples, followed by *Pseudomonas* and *Cutibacterium*. Several genera were present in both groups, including *Porphyromonas*, *Prevotella*, *Flavobacterium*, and *Actinomyces*, whereas *Bacillus*, *Aeromonas*, *Microbacterium*, *Streptococcus*, *Vibrio*, and *Brevibacterium* were detected only in NAG samples, albeit at low relative abundances. This pattern indicates that, unlike AGIM, where profiles of lower diversity and, in some cases, single-genus dominance prevailed, NAG maintained a more complex and variable community structure among individuals, with greater coexistence of genera (Figure 5).

**Figure 6:**
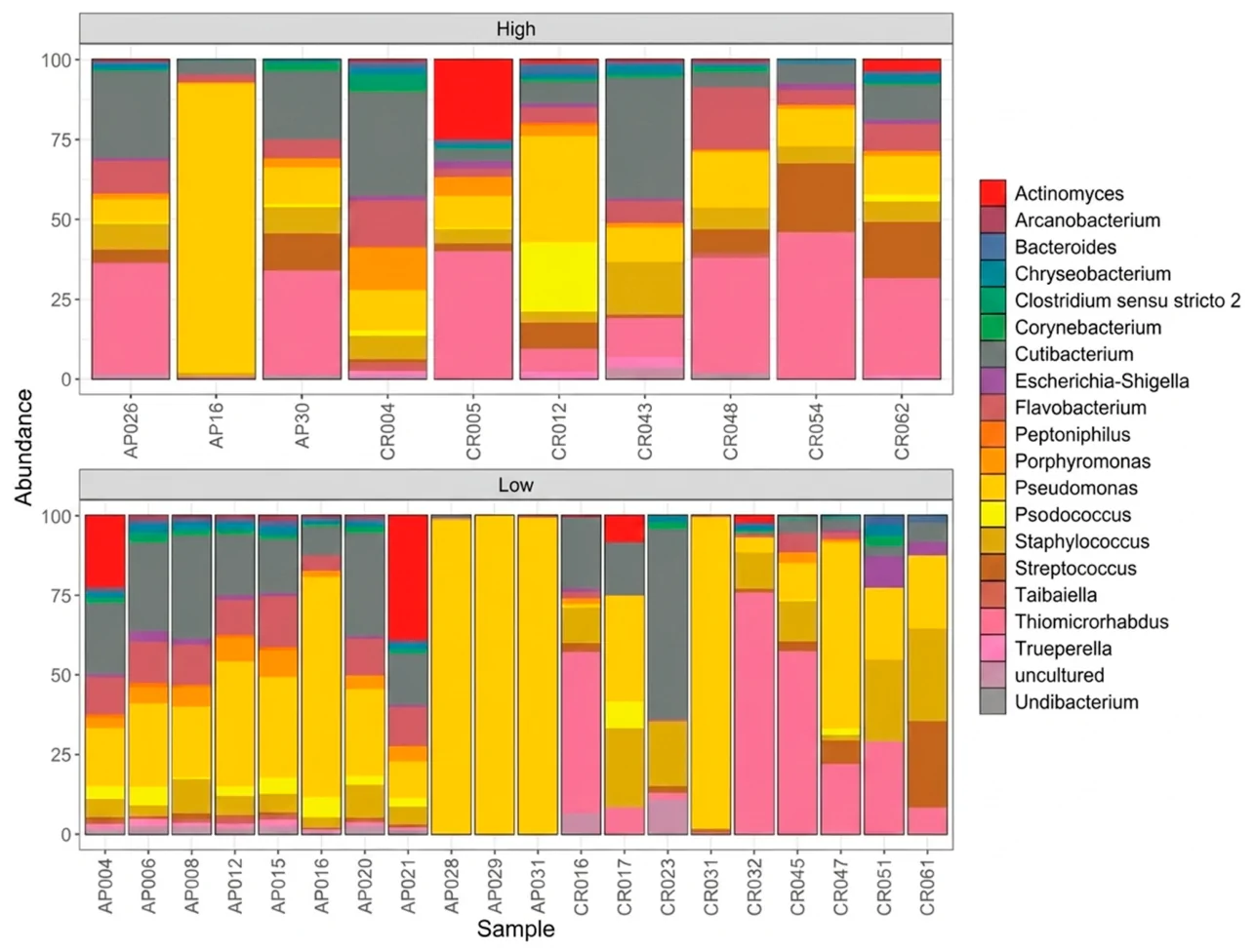
The relative abundance of the 20 most abundant bacterial genera according to *Helicobacter* categorization. Patients with a high *H. pylori* load (High, **upper panel**) and a low *H. pylori* load (Low, **lower panel**). Each bar represents an individual sample, and colors indicate the corresponding bacterial genus.

In the group with high *Helicobacter* categorization, marked variability in bacterial composition was observed among samples, reflecting notable heterogeneity in the structure of the gastric microbiota. No consistently dominant genus was identified, except in sample AP16, in which *Pseudomonas* was predominant. Several samples showed a high contribution of *Thiomicrorhabdus* and *Cutibacterium*, while genera such as *Actinomyces, Porphyromonas, Flavobacterium,* and *Streptococcus* were present in lower proportions. In contrast, the group with low *Helicobacter* categorization showed a less heterogeneous compositional pattern. In particular, *Pseudomonas* exhibited marked dominance in some samples (AP028, AP029, AP031, and CR031), followed by *Thiomicrorhabdus* and *Cutibacterium* in variable proportions. Additionally, other genera such as *Flavobacterium, Actinomyces, Staphylococcus,* and *Streptococcus* were detected at lower abundances. These results suggest that a lower *Helicobacter* load may be associated with a relatively more stable gastric microbiota, with dominant genera that recurred consistently across samples (Figure 6).

Alpha diversity (Shannon, Simpson, and ACE indices) and beta diversity analyses were performed at the genus level, which represents the highest taxonomic resolution of the dataset. These analyses were conducted according to each of the evaluated variables (lesion group and *Helicobacter* categorization). To determine significant differences between groups, appropriate statistical tests were applied according to data distribution. For the group variable, no statistically significant differences were identified among the analyzed indices (*p* > 0.05). In contrast, for the *Helicobacter* variable, significant differences were observed in the Simpson (*p* = 0.0276) and Shannon (*p* = 0.0083) indices, whereas the ACE index showed no variation between categories (*p* = 0.9928) (Table 2). These findings suggest that the presence of *Helicobacter* is associated with changes in gastric microbiota diversity, particularly in the community structure and in the relative distribution of the taxa present.

Alpha diversity analysis using the ACE index revealed no significant differences in the estimated richness of bacterial genera between the AGIM and NAG groups. The AGIM group showed greater dispersion in individual values, whereas the NAG group exhibited a more concentrated distribution within a narrow interval. However, the medians were similar and the interquartile ranges overlapped broadly, indicating that estimated richness was comparable between the two groups (*p* = 0.71616).

Regarding the Shannon index, the distributions of both groups were similar, with close medians and marked symmetry in the violin plots. The overlap of the boxes and comparable dispersion of individual data points suggest that the structure of bacterial communities, in terms of genus richness and relative distribution, did not differ notably between AGIM and NAG. These patterns are consistent with the absence of statistically significant differences between groups (*p* = 0.5267).

**Figure 7:**
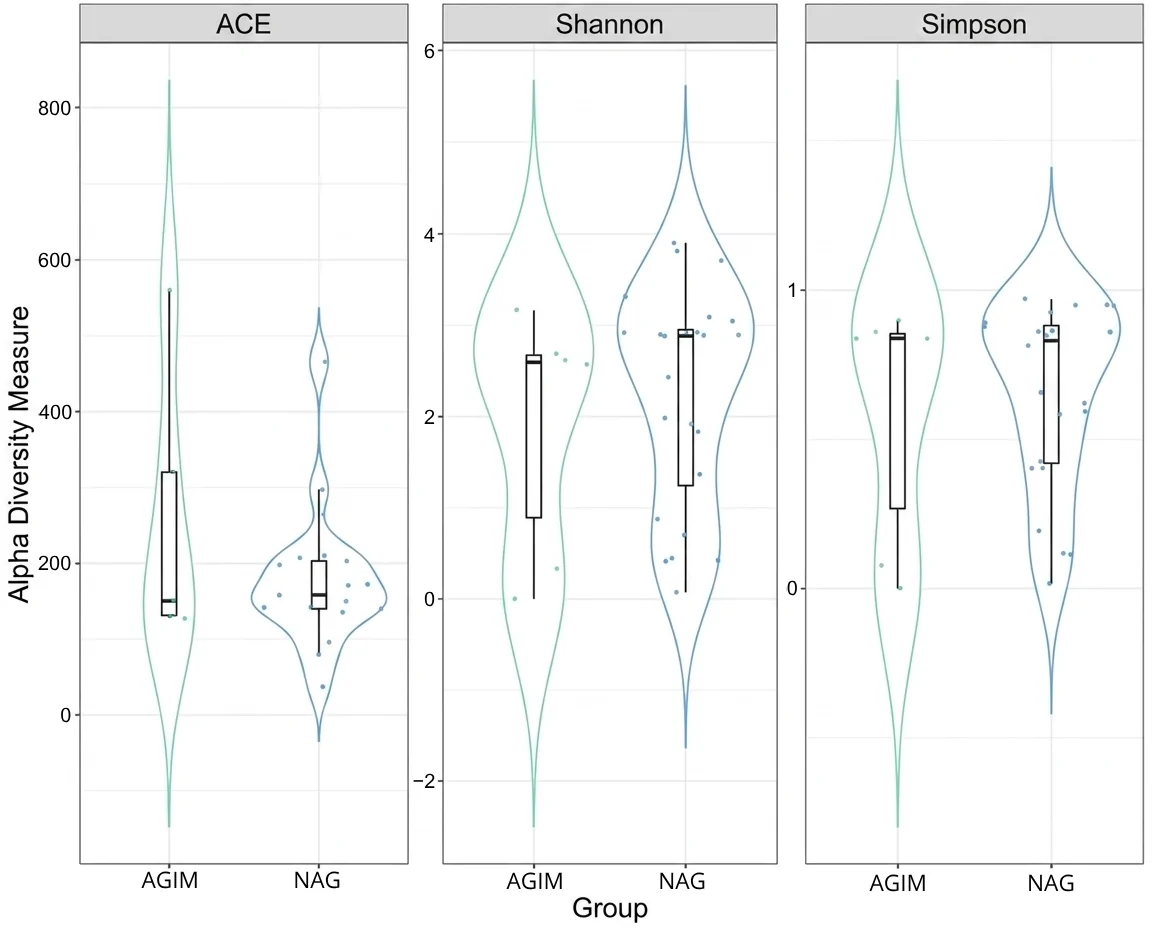
Comparison of alpha diversity (ACE, Shannon, and Simpson indices) between groups. Indices were calculated at the genus level and are represented by violin plots with overlaid box plots and individual data points.

Consistent with this, the Simpson index supported these findings. The distributions of AGIM and NAG overlapped considerably, with very close medians and comparable dispersion between groups, indicating that the dominance of certain genera and the evenness of the abundance distribution did not differ significantly between groups (*p* = 0.3739) (Figure 7).

Alpha diversity analysis using the ACE index revealed similar distributions between the high and low *Helicobacter* categories. Both groups showed comparable medians and overlapping interquartile ranges, although the low category exhibited greater variability, evidenced by a wider dispersion of individual data points. These patterns indicate the absence of notable differences in estimated genus-level richness between categories (*p* = 0.9928).

Regarding the Shannon index, samples categorized as high tended to present higher values and lower dispersion compared with the low group, suggesting a relatively more even microbial community. Nevertheless, given the partial overlap of interquartile ranges, the statistical significance of this difference should be interpreted in light of the obtained *p*-value (*p* = 0.008319).

**Figure 8:**
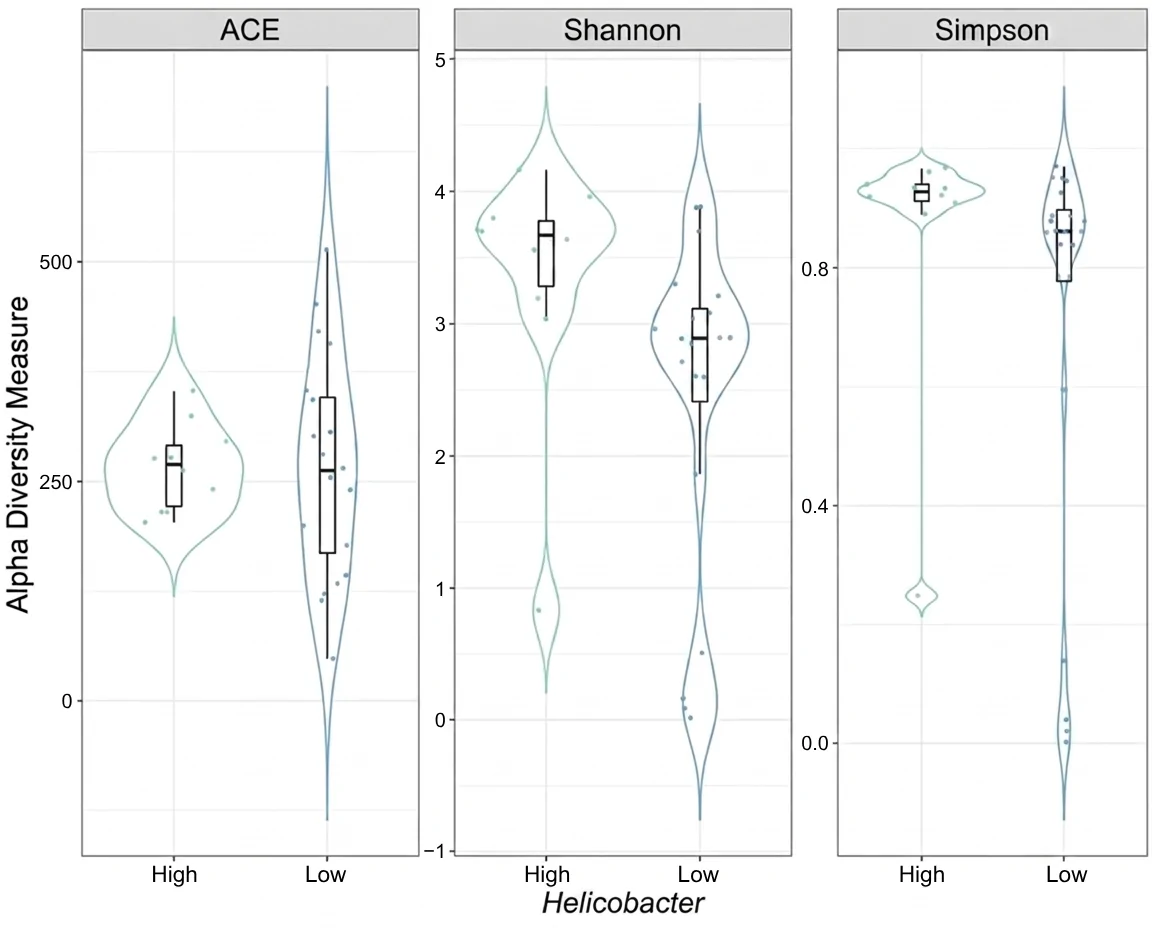
Comparison of alpha diversity (ACE, Shannon, and Simpson indices) according to *Helicobacter* categorization. Observed differences were evaluated using parametric or non-parametric tests, as appropriate.

Consistently, the Simpson index showed that samples in the high group presented higher and more homogeneous values, indicating greater diversity with a lower dominance of individual taxa. The low group exhibited a more heterogeneous distribution, with values ranging from low levels to values comparable to those of the high group, and a lower median (*p* = 0.0276). Together, the Shannon and Simpson indices suggested that the greater relative abundance of *Helicobacter* is associated with differences in bacterial community structure (Figure 8).

Beta diversity was evaluated using principal coordinates analysis (PCoA) based on the Bray—Curtis dissimilarity index. Permutational analysis of variance (PERMANOVA) based on the Bray—Curtis distance was used to assess the beta diversity of microbial communities (Table 3).

Significant differences in microbial composition were observed according to *Helicobacter* categorization (*p* = 0.012). No significant differences were found for the group variable.

**Figure 9:**
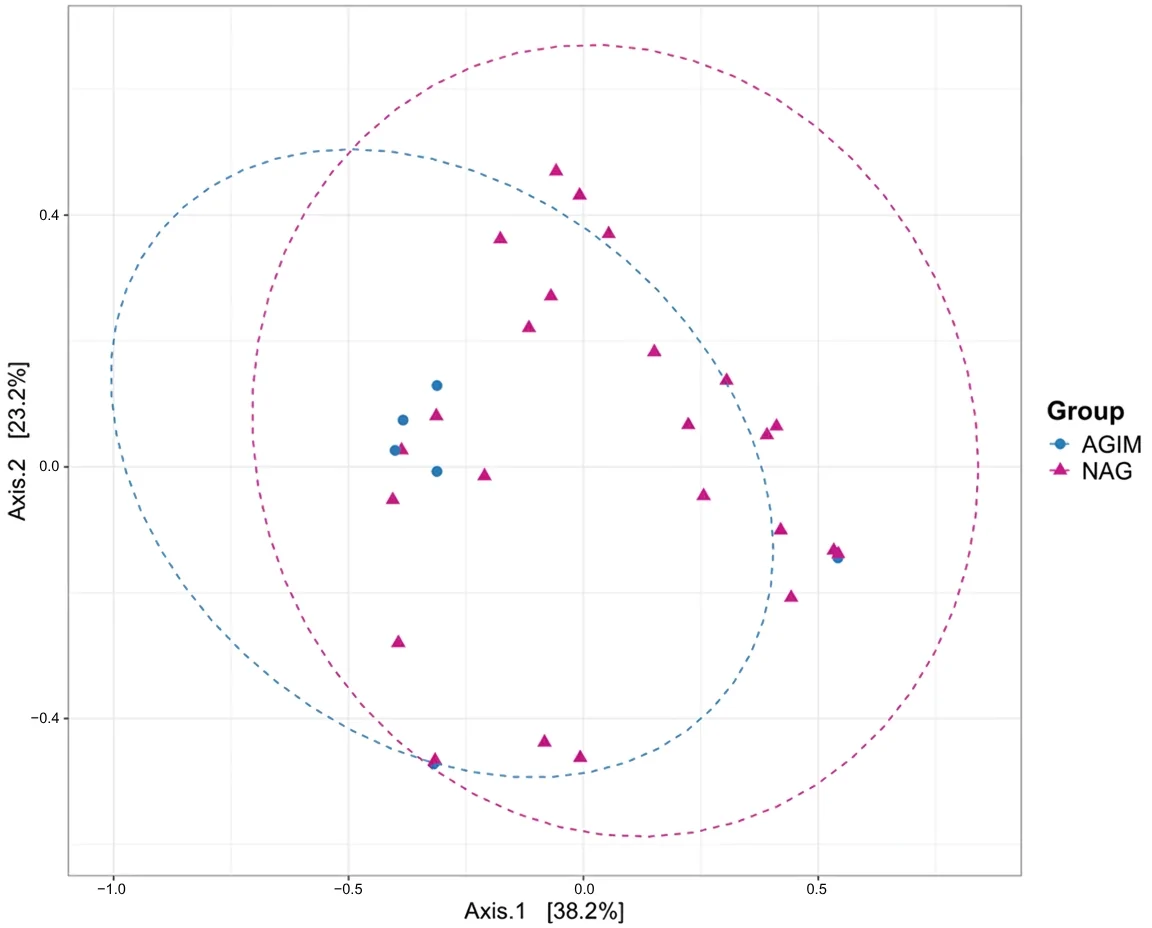
Principal coordinates analysis (PCoA) based on Bray–Curtis distance according to group type. Each point represents an individual sample and ellipses indicate the 95% confidence interval for each group.

PCoA based on Bray–Curtis distance showed that the first two axes explained 61.4% of the total variation in microbial composition (Axis.1: 38.2%; Axis.2: 23.2%). Although a tendency toward separation between groups was observed, the 95% confidence ellipses overlapped broadly, indicating that the centroids of two groups did not differ significantly in ordination space (PERMANOVA, *p* = 0.104). Furthermore, the NAG group showed a greater dispersion of samples, reflecting a more heterogeneous microbial composition, whereas the AGIM group exhibited more compact clustering, consistent with more similar microbial communities among its members. These results suggest that although some tendency toward differentiation according to gastritis type was present, within-group variability limited the definition of a distinctive compositional pattern between groups (Figure 9).

PCoA showed that the first two axes explained 62.9% of the total variation in microbial composition (Axis.1: 39%; Axis.2: 23.9%). Unlike the pattern observed according to gastritis type, the high and low *Helicobacter* categorization revealed a separation between groups. The high *Helicobacter* group showed a more compact distribution, suggesting more similar microbial communities among its members. In contrast, the low *Helicobacter* group exhibited greater dispersion, indicating a more heterogeneous microbial composition.

**Figure 10:**
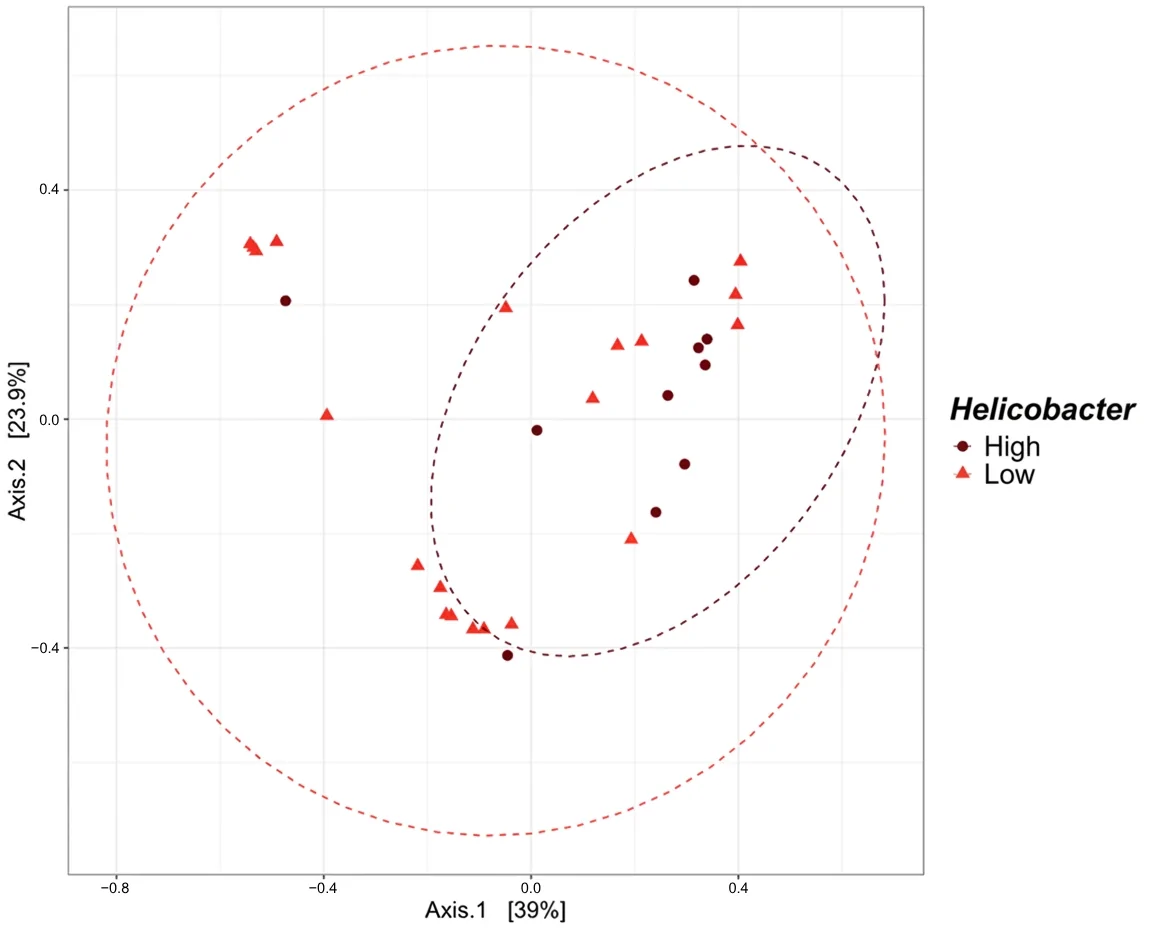
PCoA based on Bray–Curtis distance according to *Helicobacter* categorization. Significant differences in microbial composition associated with this variable were observed (*p* = 0.012).

Additionally, the 95% confidence ellipses showed limited overlap and differentiated centroids, confirming a statistically significant separation between groups (PERMANOVA, *p* = 0.012). These results suggest that the relative abundance of *Helicobacter* may be a determining factor in the structure of the gastric microbiota and was associated with changes in the overall composition of the bacterial community (Figure 10).

**Figure 11:**
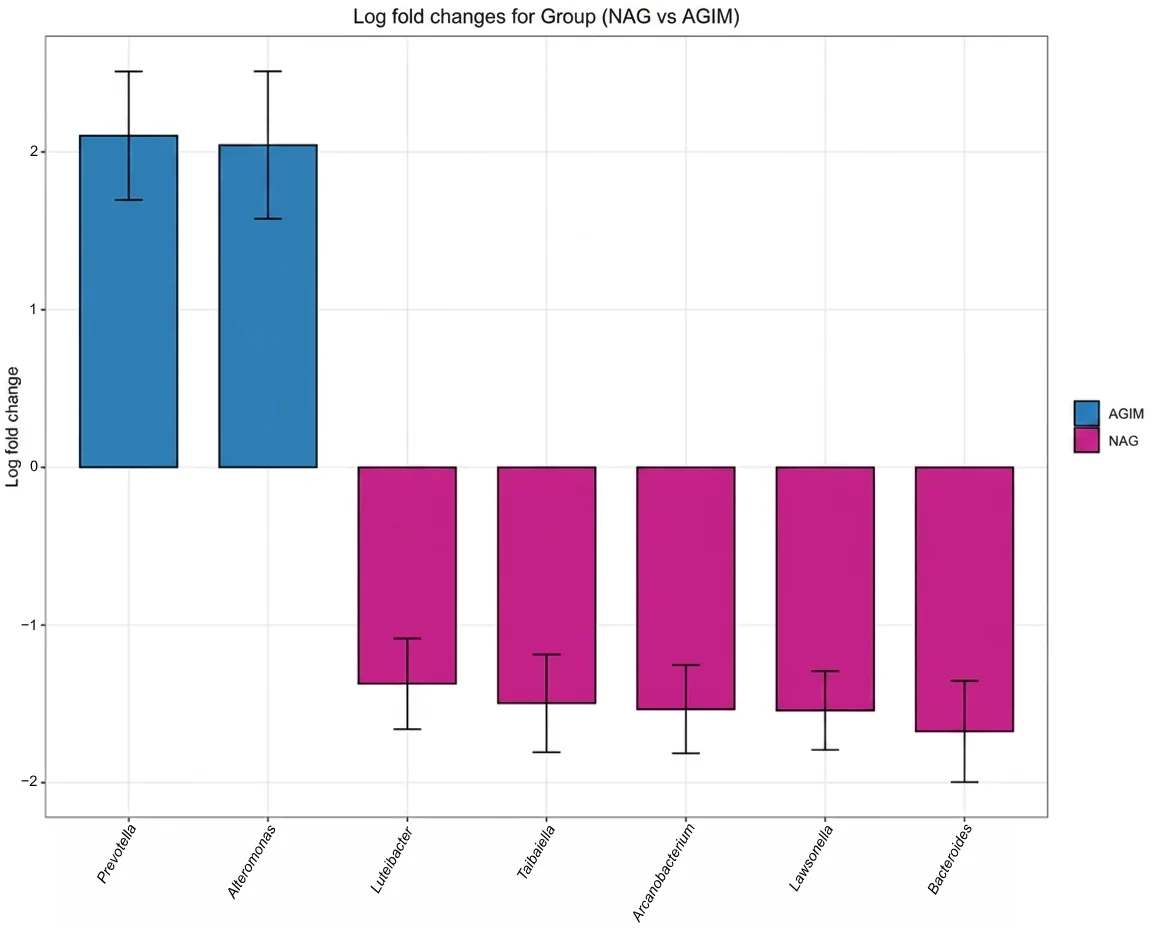
Differentially abundant genera identified by ANCOM-BC2 according to group type. Estimated log fold change values are shown for taxa with significant differences (*q* < 0.05).

Differentially abundant genera were detected in relation to group type and *Helicobacter* categorization. Seven differentially abundant genera were identified between groups (*q* < 0.05). *Prevotella* and *Alteromonas* showed positive log fold change values with confidence intervals not overlapping zero, indicating significant enrichment in AGIM. In contrast, *Luteibacter*, *Taibaiella*, *Arcanobacterium*, *Lawsonella*, and *Bacteroides* presented negative log fold change values, indicating greater relative abundance in NAG. These results reflect a differential microbiota profile associated with gastritis type (Figure 11).

**Figure 12:**
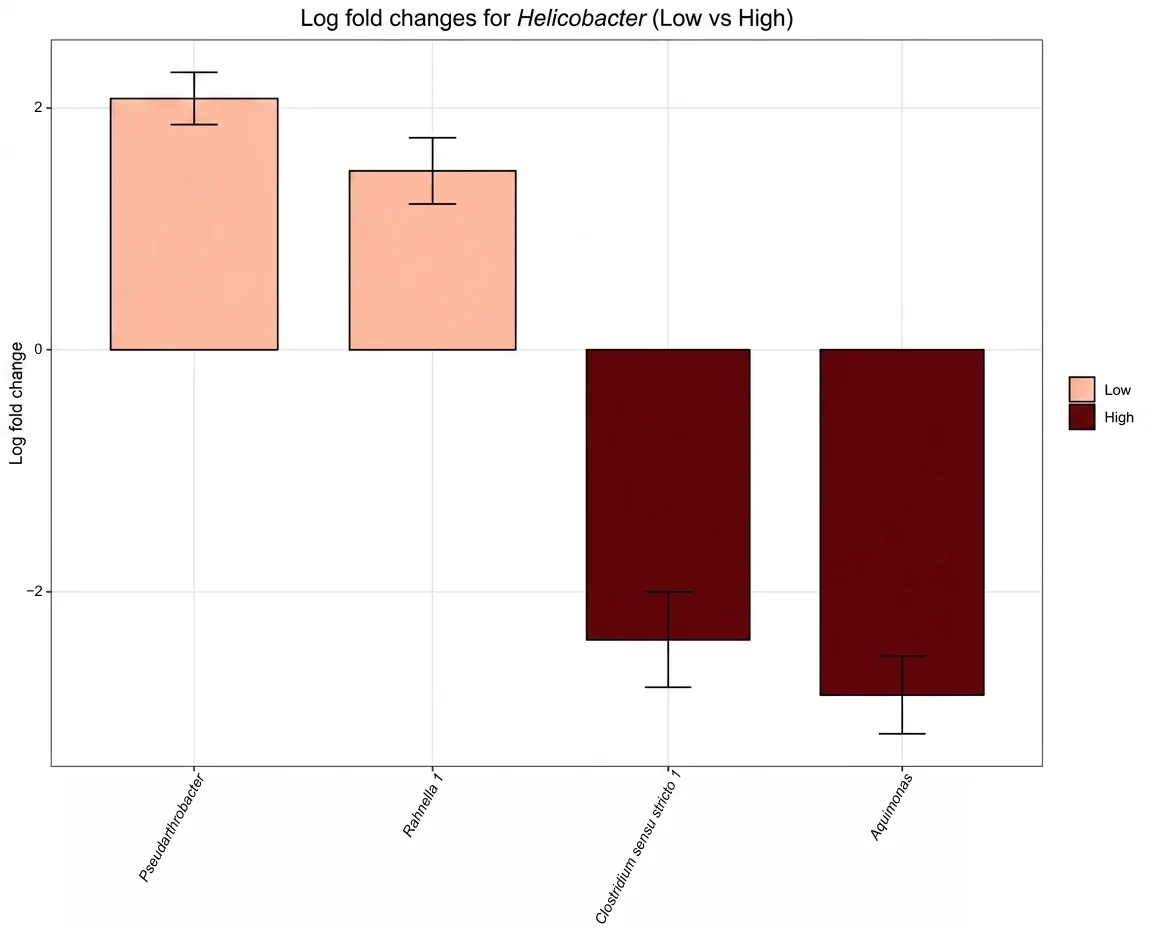
ANCOM-BC2 results for the *Helicobacter* variable. Genera with significant differences in the adjusted relative abundance between categories of this variable are shown (*q* < 0.05).

Significant differences in the relative abundance of bacterial genera were identified between *Helicobacter* categories. In the low group, *Pseudarthrobacter* and *Rahnella* presented positive log fold change values with confidence intervals not including zero, indicating greater adjusted abundance relative to the high category. In contrast, *Clostridium sensu stricto 1* and *Aquimonas* showed negative log fold change values, indicating greater relative abundance in the high category. These results suggest a differential microbiota pattern according to *Helicobacter* load, with specific taxa associated with each category (Figure 12).

Differential abundance analysis performed with DESeq2 identified bacterial genera with significant differences according to *Helicobacter* categorization. No significant differences were observed with respect to group type.

**Figure 13:**
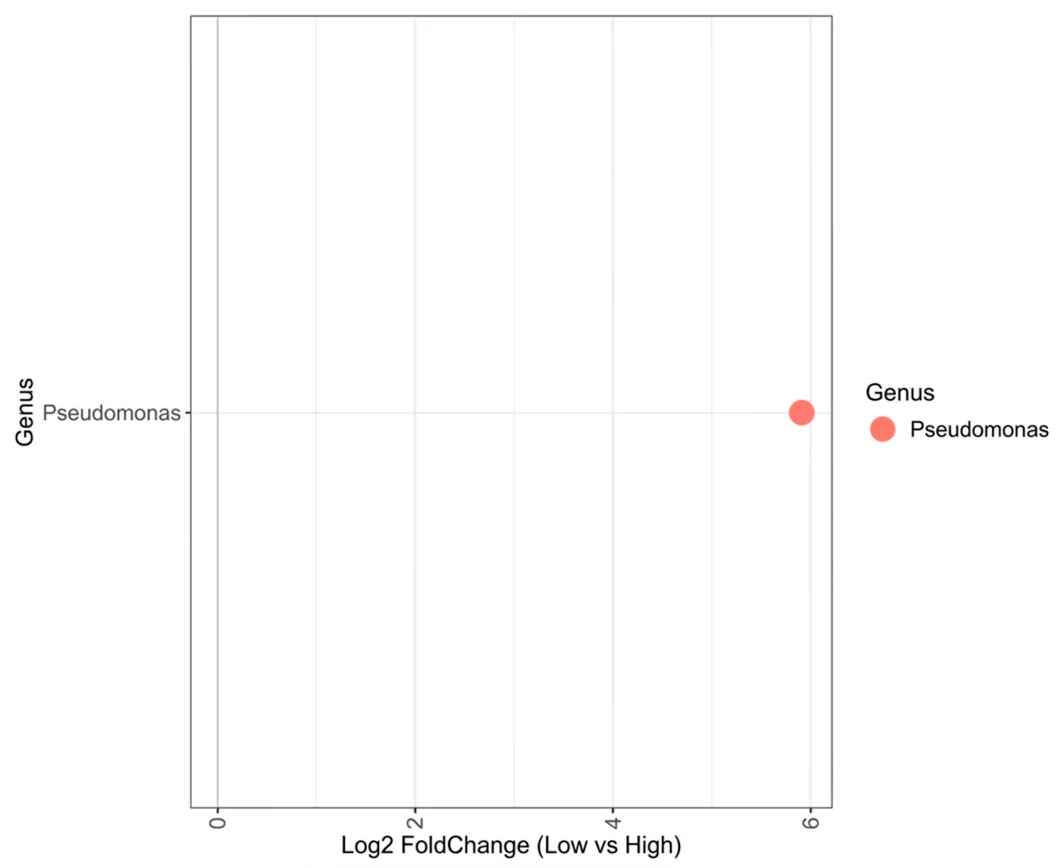
Differentially abundant genera according to *Helicobacter* categorization, identified by DESeq2. Changes in relative abundance (log2 fold change) are shown for genera with significant differences (adjusted *p* < 0.01).

The genus *Pseudomonas* was the only taxon that exceeded the significance threshold, showing greater abundance in the low group (log2 fold change ≈ 6, adjusted *p* < 0.01), representing an approximately 64-fold increase in its relative abundance. This result indicates a differential role of this genus in the gastric microbiota of individuals with lower *Helicobacter* colonization (Figure 13).

## 3. Discussion

This study characterized the composition and diversity of the gastric microbiota in patients from a region with a high risk of gastric cancer in Nariño, Colombia, providing one of the first metagenomic profiles based on long-read sequencing using the Oxford Nanopore MinION platform in this population. Sequencing enabled the identification of 912 bacterial genera distributed across 46 phyla, reflecting the broad taxonomic resolution achievable through *16S* rRNA gene amplification. This resolution is a key advantage of long-read technologies, which have been shown to substantially improve taxonomic classification accuracy (from 65% to 96.5%) [24,25]. This capability is particularly relevant to the study of the gastric microbiota, given the acidic environment of the stomach, which limits bacterial colonization and taxa detection [26].

Diversity analyses showed that gastric community structure was associated with *Helicobacter* relative abundance rather than with histological lesion type. Samples categorized as having high *Helicobacter* abundance exhibited significantly greater alpha diversity (Shannon: *p* = 0.008; Simpson: *p* = 0.028) and distinct beta diversity (PERMANOVA, Bray–Curtis: *p* = 0.012) compared with the low abundance category. In contrast, no statistically significant differences in alpha or beta diversity were detected between the NAG and AGIM groups. These results suggest that *Helicobacter* relative abundance is associated with gastric microbiota structure, irrespective of the histological stage of the mucosa; the cross-sectional design, however, precludes establishing the direction of this relationship.

A plausible explanation for the association between high *Helicobacter* abundance and increased microbial diversity is that *H. pylori* colonization modifies the gastric environment in ways that attenuate conditions adverse to other microorganisms [27]. The mechanism most frequently proposed to explain this reconfiguration is ammonia production mediated by urease subunits A and B, which hydrolyze urea to carbon dioxide and ammonia, locally raising the pH and potentially enabling colonization by bacteria that would otherwise be excluded from the gastric environment [21,28,29]. This alkalinizing effect has been reported to be amplified during chronic infection, in which *H. pylori* disrupts interactions among parietal, G, ECL and D cells, compromising the regulation of gastric acid secretion and promoting progressive hypochlorhydria, which may broaden the ecological space available for taxa not adapted to an acidic environment [30,31]. However, gastric pH and acid secretion were not measured in the present study; therefore, these physiological changes cannot be demonstrated directly or established as the mechanism underlying the higher diversity observed in the high *Helicobacter* category.

When considering *Helicobacter* categorization, high colonization was associated with dominance of the phylum *Proteobacteria*, reduced relative abundance of *Firmicutes*, *Actinobacteriota*, and *Bacteroidota*, and greater alpha diversity. In contrast, samples with low *Helicobacter* abundance displayed greater compositional heterogeneity and greater interindividual variability, potentially reflecting the influence of host-specific factors in the absence of the modulatory effect of *H. pylori*. These patterns are consistent with previous studies reporting *Proteobacteria* dominance regardless of infection status, but with markedly different proportions depending on *H. pylori* load [31,32,33]. In *H. pylori*-positive individuals, in addition to the pathogen itself, other genera such as *Haemophilus, Serratia, Neisseria,* and *Stenotrophomonas*, belonging to the phylum *Proteobacteria*, constitute the dominant phylum of the gastric community, with lower representation of *Actinobacteria*, *Bacteroidetes*, and *Firmicutes* [34]. This pattern agrees with the present findings and has been reported across populations with varying infection prevalence [35,36,37,38].

Differential abundance analysis supported this interpretation. ANCOM-BC2 identified *Clostridium sensu stricto 1* and *Aquimonas* as significantly more abundant in the high *Helicobacter* category, whereas *Pseudarthrobacter* and *Rahnella* were enriched in the low *Helicobacter* category. Notably, *Pseudomonas* enrichment in the low *Helicobacter* abundance group was confirmed by DESeq2 with a log2 fold change of approximately 6. This pattern is consistent with previous reports identifying *Pseudomonas* as a consistently enriched taxon in gastric environments with low or absent *H. pylori* colonization [18,39]. At the species level, however, *Pseudomonas stutzeri* has also been detected in *H. pylori*-positive individuals, suggesting the capacity to adapt to the gastric microenvironment altered by infection. The displacement of *Pseudomonas* in the high colonization group is consistent with mechanisms of competitive exclusion driven by *H. pylori* virulence factors, complemented by specific microniches in the gastric glands where founder bacteria actively prevent colonization by new microorganisms, as well as the influence of pH and oxygen levels on bacterial community structure [40,41,42].

When analyzing group type, *Proteobacteria* and *Actinobacteriota* predominated in most samples, which is consistent with previous reports, in which these phyla, together with *Firmicutes* and *Bacteroidota,* constitute the principal components of the gastric microbiota [26,43,44]. In particular, *Proteobacteria* showed a numerically greater relative abundance in NAG compared with AGIM. This observation contrasts with previous findings reporting a lower representation of this phylum in non-atrophic gastritis (34.9%) than in severe atrophic gastritis (40.8%), a discrepancy that may be related to differences in sample size, sequencing platform, and the geographic origin of the populations studied [45].

Regarding genus-level composition, both groups shared dominant taxa but differed in compositional heterogeneity. In AGIM, fewer genera were detected in some samples, along with marked dominance of taxa such as *Pseudomonas, Helicobacter*, or *Cutibacterium* depending on the sample, a profile compatible with dysbiosis associated with chronic inflammation and alterations of the gastric environment [26], and consistent with previous reports identifying *Helicobacter, Streptococcus*, and *Lactobacillus* among the most abundant genera in precancerous stages, including atrophic gastritis. In contrast, NAG samples showed a more heterogeneous community profile, with genera such as *Bacillus, Aeromonas, Microbacterium, Vibrio*, and *Brevibacterium* detected only in this group, albeit at low relative abundances, which may reflect an ecologically more open mucosal community amenable to colonization [46]. One mechanism proposed to explain restricted colonization in advanced gastritis is the progressive replacement of the gastric mucins MUC5AC and MUC6 by MUC2, characteristic of the intestinal epithelium, which reduces the capacity of the mucosa to sustain stable microbial communities [47,48,49]. Mucin expression was not evaluated in this cohort, so this remains a hypothesis rather than an explanation of the present findings.

Although no statistically significant differences in alpha or beta diversity were detected between AGIM and NAG, differential abundance analysis using ANCOM-BC2 identified seven genera with significantly different relative abundances between groups (*q* < 0.05). *Prevotella* and *Alteromonas* exhibited greater abundance in AGIM, whereas *Luteibacter, Taibaiella, Arcanobacterium, Lawsonella,* and *Bacteroides* were more abundant in NAG. The enrichment of *Prevotella* in AGIM contrasts with previous reports describing its reduction in atrophic gastritis relative to healthy controls [27,50].

Previous research in the department of Nariño has documented geographic differences in gastric microbiota composition associated with GC risk. Using *16S* rRNA gene deep sequencing, a previous study found geographic origin to be the main factor correlated with community structure between high- and low-risk municipalities (AMOVA, Jaccard, *p* = 0.004), with distinct differentially abundant taxa in Túquerres and Tumaco; notably, no correlation was detected with *H. pylori* phylogeographic type or cag pathogenicity island (*cagPAI*) status, suggesting that community differences are largely independent of the characteristics of the infecting strain [51]. Similarly, whole-metagenome sequencing of antral biopsies from Túquerres and Tumaco revealed differences in taxonomic and functional profiles according to infection status and risk group [52].

In a population from Nariño with contrasting gastric cancer risk, profiles from patients with non-atrophic gastritis showed a lower relative abundance of *Epsilonproteobacteria* together with greater metagenomic diversity, whereas profiles from patients with atrophic gastritis with intestinal metaplasia were dominated by this class; notably, histological stage itself did not significantly affect diversity (*p* > 0.05); diversity was instead reduced by *H. pylori* infection in the antrum (*p* = 0.005) [53].

GC is a multifactorial disease whose development involves the interplay of environmental, genetic, and infectious factors that determine the risk of progression toward premalignant lesions [54]. Phylogenomic analysis of the *H. pylori* core genome has identified two local subpopulations, hspColombia_Andes, predominant in Andean municipalities with GC mortality rates of between 20 and 50 per 100,000 inhabitants, and hspColombia_PacificCoast, associated with the low-risk coastal region. That analysis also reported a positive correlation between altitude and GC mortality across the 64 municipalities of the department (Spearman, *p* < 0.05) [55]. Andean strains more frequently carry high-virulence genotypes (*vacA* s1m1, *cagA*-positive) and elicit a Th1 response, whereas coastal strains carry low-virulence genotypes (*vacA* s2m2, *cagA*-negative) in a Th2 context, a contrast interpreted as a shift from mutualism to pathogenicity [56,57]. Host genetic background contributes as well: the *IL-10*−1082A/G genotype was associated with a more than five-fold increased risk of atrophic gastritis in Túquerres (OR = 5.45; 95% CI, 1.0–30.59) but not in Tumaco, and the *IL-1B*−511T/T and *IL-10*-1082AA genotypes, linked to a pro-inflammatory profile, were more frequent in the Andean population [13,58].

Dietary patterns in the Andean region, characterized by high salt intake and low consumption of fruit, vegetables, antioxidants, and seafood, in contrast to the Pacific coast diet, together with helminth co-infection, prevalent along the coast, have been associated with differences in the gastric inflammatory response [6,55,59]. The Andean diet favors increased *cagA* gene expression and sustained chronic inflammation, whereas the coastal diet appears to confer a protective effect against carcinogenesis, and helminth co-infection promotes a shift toward a Th2 response that reduces gastric damage, in contrast to the *IL-10* and *IL-1β* polymorphisms described in the Andean zone, which favor a persistent Th1 response. All of these factors may contribute to gastric microbiota dysbiosis and the dominance of taxa associated with premalignant lesions [60].

This study characterized the baseline gastric microbiota at precancerous stages in a population with one of the highest reported incidences of gastric cancer worldwide [4,7], providing a reference point for the identification of microbial biomarkers of risk and guiding early endoscopic surveillance strategies. Genera with differential abundance provide candidate taxa for future research as potential co-carcinogenic or protective agents. Shotgun metagenomic and metabolomic studies could elucidate whether these taxa are involved in nitrosamine production, chronic inflammatory signaling, or mucosal barrier disruption, all of which are mechanistically linked to gastric carcinogenesis [19].

Several limitations must be acknowledged. The small sample size (NAG *n* = 24; AGIM *n* = 6) limits statistical power and may have precluded the detection of significant diversity differences between lesion types. The group imbalance, inherent in retrospective clinical availability, should be addressed in prospective studies with balanced cohorts. The cross-sectional design precludes causal inference regarding the directionality of the relationship between *Helicobacter* abundance and microbiota structure. Intragastric pH, acid secretion, and urease activity were not assessed; therefore, the mechanistic interpretation involving urease-mediated ammonia production and hypochlorhydria remains inferential and based on published evidence. Finally, variables known to differ across the risk regions of Nariño, including *H. pylori* virulence genotypes, dietary patterns, and helminth co-infection, were not characterized in this cohort, and their potential contribution to the observed variability could not be evaluated.

This study lacks independently sequenced negative controls for assessing potential contamination. During the amplification step, a negative PCR control containing DNA-free water and a positive control consisting of DNA from a bacterial isolate were included to verify the absence of nonspecific amplification and the adequacy of the PCR, respectively; however, these controls were not processed as independent sequencing libraries. Therefore, potential contaminant signals were assessed indirectly through read quality filtering, relative abundance, and the consistency of taxonomic profiles across samples. Only high-quality reads (Phred Q ≥ 15) were retained for downstream analyses and taxonomically classified using Kraken against the SILVA database, while low-abundance taxa (<1% relative abundance) were interpreted cautiously and were not considered individually as biologically relevant findings. Although this threshold does not distinguish contamination from true low-abundance microorganisms, it provides a conservative approach to minimize overinterpretation of minor taxonomic signals. Because gastric biopsies may contain relatively low microbial biomass, the potential contribution of reagent- or environment-derived bacterial DNA cannot be completely excluded, particularly for taxa detected at low abundance. Consequently, the main conclusions of this study were based primarily on predominant and consistently detected taxa. Future studies should include negative controls for DNA extraction, PCR amplification, and library preparation and process them through sequencing in parallel with biological samples to enable direct identification and appropriate filtering of potential contaminants.

## 4. Materials and Methods

**High-risk area**

**Figure 14:**
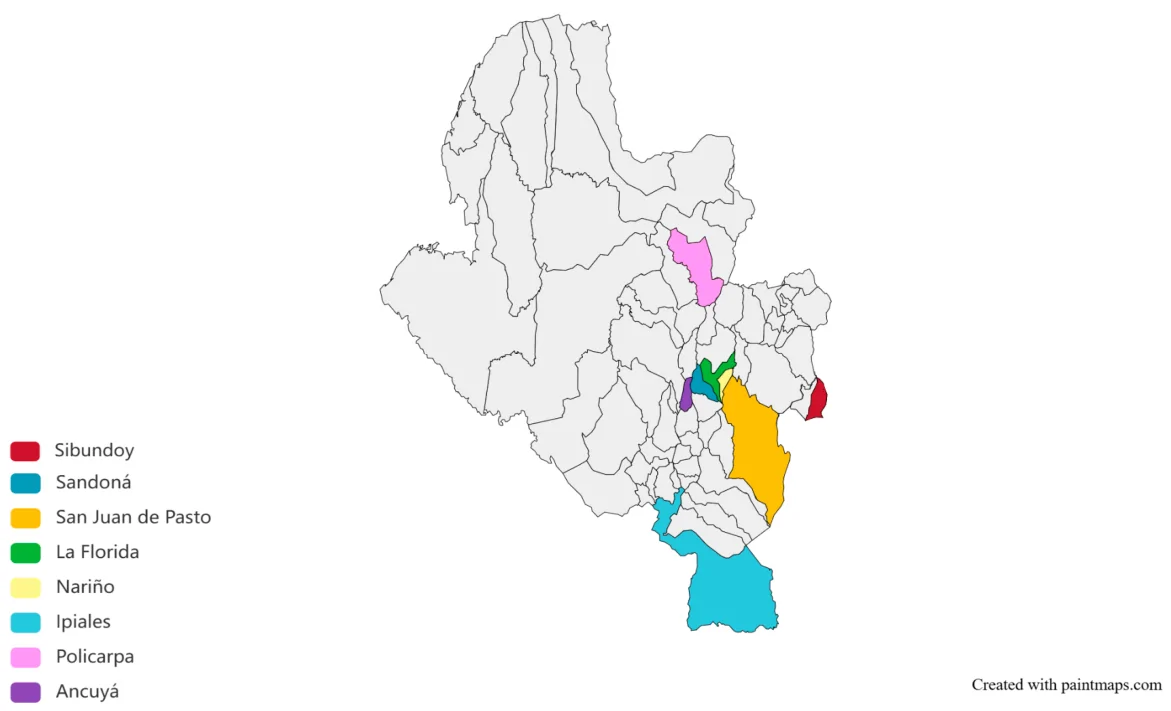
A map of the study area: the origin of patients at high risk of gastric cancer in Nariño. The colored municipalities represent the geographic distribution of cases included in the study, comprising municipalities within the department of Nariño, Colombia, as well as one neighboring municipality in the department of Putumayo: Sibundoy (dark red), Sandoná (blue), San Juan de Pasto (orange), La Florida (green), Nariño (yellow), Ipiales (light blue), Policarpa (pink), and Ancuya (purple). All remaining municipalities are shown in gray. The map was obtained from Paintmaps https://www.paintmaps.com (accessed on 29 August 2026) for Nariño and Putumayo, Colombia, South America. Map geometries were obtained from Who’s On First https://whosonfirst.org/docs/licenses/ (accessed on 29 August 2026).

The department of Nariño is located in the southwestern region of Colombia and has a population of approximately 1,800,000 inhabitants distributed across 64 municipalities, of which 81.7% of the population resides in the Andean zone, encompassing 55 municipalities. This territory of 32,265 km^2^ comprises three geographically distinct zones with respect to the incidence of GC. Particularly relevant are two regions classified as red zones, located in the northern and southwestern parts of the department, which have among the highest incidence rates worldwide (150 cases per 100,000 inhabitants), accompanied by a high prevalence of precancerous gastric lesions and *H. pylori* infection. Patients included in this study were from high-incidence Andean municipalities, including La Florida, Pasto, Policarpa, Nariño, Ipiales, Ancuya, Sandoná, and Sibundoy (Figure 14).

**Patients and sample collection**

Samples were collected from 30 patients from the municipalities described above, without sex restriction, of whom 24 had a histological diagnosis of NAG and 6 had AGIM. Patients were selected according to the following inclusion criteria: individuals over 18 years of age presenting with dyspeptic symptoms, in a stable medical and physical condition, who voluntarily agreed to participate in the study, provided written informed consent, and completed a survey addressing sociodemographic, health-related, family, and environmental factors. Exclusion criteria were a diagnosis of cancer, chronic transmissible diseases, pregnancy, ongoing anticoagulant therapy, or the use of proton pump inhibitors or antibiotics targeting *H. pylori* infection in the month preceding endoscopy.

**Biopsy collection by endoscopy**

A gastroenterologist obtained four samples per patient, two from the antrum and two from the gastric body, which were preserved in formalin–saline solution. An additional four biopsies were collected for microbiological analysis, two from the antrum (lesser curvature) and two from the body (greater curvature and fundus). These samples were preserved in thioglycolate (Merck^®^, Darmstadt, Germany) supplemented with glycerol (Promega^®^, Madison, WI, USA) in pre-labeled NUNC^®^ tubes (Thermo Fisher Scientific, Roskilde, Denmark), maintaining a microaerophilic environment to support *H. pylori* viability. Biopsies were immediately snap-frozen in liquid nitrogen and transported to the Microbiology and Molecular Biology Laboratory of the Universidad de Nariño, where they were stored at −70 °C in a Revco^®^ ULT 2586-5-A37 ultra-low temperature freezer (Thermo Fisher Scientific, Asheville, NC, USA) [61].

**DNA extraction and amplification of the** ***16S*** **rRNA**

DNA was extracted from gastric biopsy samples using the DNA Isolation Kit for Cells and Tissues (Roche Diagnostics GmbH, Mannheim, Germany), following the manufacturer’s instructions for tissue samples. DNA concentrations were assessed by spectrophotometry using a NanoDrop 2000/2000c instrument (Thermo Fisher Scientific, Waltham, MA, USA); A260/A280 ratios between 1.8 and 2.0 were considered optimal. For the detection of bacterial communities, conventional PCR was performed using primers 27F (5′-AGAGTTTGATCCTGGCTCAG-3′) and 1492R (5′-GGTTACCTTGTTACGACTT-3′), targeting full-length amplification of the *16S* rRNA gene to achieve higher resolution in the identification of bacterial communities [56,57]. PCR reactions were carried out in a final volume of 13 µL containing 6.25 µL of LongAmp Taq Master Mix (New England Biolabs, Ipswich, MA, USA) at a final concentration of 1×, 1.25 µL of each primer (1 µM), 2.25 µL of nuclease-free water, and 2 µL of genomic DNA. The thermal cycling profile consisted of an initial denaturation step at 94 °C for 30 s, followed by 30 cycles of denaturation at 94 °C for 30 s, annealing at 47.9 °C for 1 min, and extension at 65 °C for 1 min 15 s, with a final extension at 65 °C for 10 min. A DNA isolate from an *Enterobacteriaceae* species served as the positive control; nuclease-free water served as the negative control.

**Sequencing with Oxford Nanopore Technologies (ONTs)**

Barcoding and adapter ligation were performed using the SQK-NBD114.96 kit (Oxford Nanopore Technologies, Oxford, UK). Amplified DNA was purified using AMPure^®^ XP beads (Beckman Coulter, Brea, CA, USA), and the prepared library was loaded onto an R10.4.1 flow cell (FLO-MIN114; Oxford Nanopore Technologies, Oxford, UK). Long-read sequencing was performed using MinKNOW software v1.11.5 (Oxford Nanopore Technologies, Oxford, UK).

**Bioinformatic analysis and sequencing data processing**

For basecalling, POD5 files generated by the sequencing platform were processed using Dorado software (v2.1.1; Oxford Nanopore Technologies, Oxford, UK) with the super-accuracy model, retaining reads with a Q-score ≥ 10 [62,63]. Mean read quality, expressed as Phred score, was assessed using NanoPack (NanoPlot version 1.46.2) [64]. Taxonomic assignment was performed against the SILVA v132 *16S* rRNA database, which is widely used in microbial community studies due to its extensive coverage and manual curation [65]. Read clustering and taxonomic classification were carried out using Kraken (version 2.17.1), a k-mer-based tool that enables rapid and accurate assignment across multiple taxonomic levels [66]. Kraken outputs were subsequently visualized using Pavian (version 1.2.1), an interactive tool for graphical exploration of sample taxonomic composition. Pavian facilitates the detection of distributional patterns, cross-sample comparisons, and identification of dominant taxa through bar charts, interactive tables, and Sankey diagrams [67].

**Determination of relative bacterial abundance**

Bacterial community composition was analyzed using R v4.4.1 (https://www.r-project.org/ accessed on 15 September 2026) with several specialized packages for microbial ecology and data visualization. Taxonomic data and sample metadata were organized and processed using the phyloseq package (v1.56.0), which enables the integration of abundance matrices, taxonomic information, and associated variables into a coherent framework for downstream analyses [67,68]. To account for differences in sequencing depth across samples, taxonomic counts were normalized and transformed to relative abundances using functions from the microbiome package (v1.34.0) [69]. Stacked bar charts representing taxa distribution at multiple taxonomic levels were generated using ggplot2 and ggpubr [67]. To facilitate interpretation, taxa with a relative abundance below 1% at the phylum level were grouped into an “Others” category; at the family level, taxa below 5% were similarly grouped; and at the genus level, the 20 most abundant taxa were displayed. This approach highlighted dominant taxa and enabled comparison of their distribution across experimental groups and conditions.

Samples were stratified according to the relative abundance of the genus *Helicobacter* obtained from ONT-based taxonomic profiling. Rather than applying a predefined clinical threshold, median, or quartile-based cutoff, an unsupervised k-means clustering approach (k = 2) was applied to the relative abundance values. This procedure generated two clusters comprising 20 samples with low *Helicobacter* abundance and 10 samples with high abundance. The resulting cluster centroids were 6.76% and 78.22%, respectively. In this one-dimensional clustering, the corresponding decision boundary between the two centroids was approximately 42.49% relative abundance. This value therefore represents a data-derived clustering boundary rather than a predefined biological or clinical cutoff.

**Evaluation of alpha and beta diversity**

Alpha diversity indices (Shannon, Simpson, and ACE) were estimated from the phyloseq object using the estimate_richness function. These indices were visualized using violin plots overlaid with box plots and individual data points, generated with the ggpubr (v1.0.0), ggplot2 (v4.0.3), and ggforce (v0.5.0) packages [70]. Plots displayed index distributions by analytical variable, with differentiated color coding to facilitate interpretation. Prior to between-group comparisons, normality was assessed for each index using the Shapiro–Wilk test. Depending on data distribution and the number of groups, either parametric (Student’s *t*-test or ANOVA) or non-parametric (Wilcoxon rank–sum or Kruskal –Wallis) tests were applied to evaluate significant differences. Beta diversity was assessed using the Bray–Curtis dissimilarity index following normalization of data to relative proportions. PCoA was performed, and the results were visualized using the plot_ordination function from the phyloseq package (v1.56.0). Confidence ellipses were added per group using stat_ellipse with a t-distribution. Differences in microbial community structure between groups were evaluated using permutational analysis of variance (PERMANOVA), implemented with the adonis2 function from the vegan package (v2.7-5) [70].

**Differential abundance analysis by study variable**

Differential abundance analysis of bacterial genera was conducted using the ANCOM-BC2 package (v2.14.0). Relative abundance matrices, taxonomy tables, and metadata were organized into phyloseq-compatible objects to ensure correct association between samples and categorical variables [71,72]. The analysis was performed at the genus level and incorporated the categorical variables defined in the study as fixed effects within the model. Filtering criteria, multiple testing correction, structural zero handling, and log fold change estimation were applied. The results were exported and interpreted by identifying taxa with significant differences between the evaluated categories.

Additionally, differential abundance analysis was performed using the DESeq2 package (v1.52.0) in R. Amplicon sequence variant (ASV) abundance matrices, taxonomy tables, and sample metadata were imported and integrated into a phyloseq object, with ASV and sample identifiers assigned as row names. A DESeq2-compatible object was generated from the phyloseq object using the phyloseq_to_deseq2 function, and a Wald test with a general design model was applied to identify significant differences in the relative abundance of taxa [70,73,74]. The results were filtered using an adjusted *p*-value threshold of 0.01 and integrated with taxonomic information for interpretation. Only taxa identified at the genus level were included in the visualization. Log2 fold change values were plotted in a scatter plot using ggplot2, highlighting genera with statistically significant differences [70]. Relative abundances and comparisons of diversity indices were analyzed according two categorical variables: lesion group (AGIM or NAG) and *Helicobacter* abundance (high and low).

## 5. Conclusions

This study characterized the gastric microbial communities of 30 patients from Nariño, Colombia, a region with one of the highest gastric cancer incidences worldwide, comprising 24 cases of non-atrophic gastritis (NAG) and 6 of atrophic gastritis with intestinal metaplasia (AGIM). Full-length *16S* rRNA gene sequencing on the Oxford Nanopore MinION platform, among the first such profiles reported for this population, revealed communities dominated by *Proteobacteria, Campylobacterota*, and *Actinobacteriota*, with *Helicobacter, Pseudomonas,* and *Cutibacterium* as the most abundant genera and marked interindividual variability.

Gastric community structure varied with the relative abundance of *Helicobacter* rather than with histological diagnosis. Samples in the high *Helicobacter* category showed significantly greater evenness and a lower dominance of individual genera, whereas estimated genus richness was comparable between categories, indicating that the observed shifts reflected how abundances were distributed rather than how many genera were present. Overall community composition also differed significantly between *Helicobacter* categories, although this variable accounted for only part of the compositional variation observed among individuals. By contrast, no significant differences in either alpha or beta diversity were detected between NAG and AGIM, suggesting that microbial variation associated with *Helicobacter* abundance was more evident than that associated with histological stage of the lesion types evaluated.

Differential abundance testing identified a small number of candidate genera. *Prevotella* and *Alteromonas* were enriched in AGIM, whereas *Luteibacter*, *Taibaiella*, *Arcanobacterium*, *Lawsonella*, and *Bacteroides* were more abundant in NAG. According to *Helicobacter* burden, *Clostridium sensu stricto 1* and *Aquimonas* were enriched in the high category, while *Pseudarthrobacter, Rahnella* and *Pseudomonas* were markedly more abundant in the low category. These taxa are candidates for future research as biomarkers of neoplastic progression or as agents with a protective or co-carcinogenic role, a hypothesis that requires confirmation in longitudinal studies with greater statistical power.

## Figures and Tables

**Table 1:** Distribution of patients by sample ID, histopathological diagnosis, age, sex, and municipality of origin.

Sample ID	Histopathological Diagnosis	Age	Sex	Municipality of Origin
CR051	Non-atrophic gastritis	30	F	Pasto
CR012	Non-atrophic gastritis	33	F	Pasto
CR031	Non-atrophic gastritis	50	F	Policarpa
CR047	Non-atrophic gastritis	62	F	Pasto
AP029	Atrophic gastritis with intestinal metaplasia	48	F	La Florida
AP015	Atrophic gastritis with intestinal metaplasia	50	M	La Florida
AP020	Atrophic gastritis with intestinal metaplasia	56	F	La Florida
AP012	Non-atrophic gastritis	54	M	La Florida
CR043	Non-atrophic gastritis	18	F	Nariño
AP006	Non-atrophic gastritis	40	F	La Florida
AP16	Non-atrophic gastritis	52	F	La Florida
AP021	Atrophic gastritis with intestinal metaplasia	50	F	La Florida
AP004	Non-atrophic gastritis	67	F	La Florida
AP008	Atrophic gastritis with intestinal metaplasia	60	M	La Florida
AP028	Non-atrophic gastritis	54	F	La Florida
AP026	Non-atrophic gastritis	68	F	La Florida
CR061	Non-atrophic gastritis	36	M	Pasto
CR062	Non-atrophic gastritis	29	M	Sibundoy
AP016	Non-atrophic gastritis	52	F	La Florida
AP031	Non-atrophic gastritis	52	F	La Florida
CR045	Non-atrophic gastritis	34	M	Pasto
CR054	Non-atrophic gastritis	30	F	Ipiales
CR005	Non-atrophic gastritis	30	F	Pasto
CR004	Non-atrophic gastritis	68	M	Pasto
AP30	Atrophic gastritis with intestinal metaplasia	44	F	La Florida
CR048	Non-atrophic gastritis	48	F	Ancuya
CR016	Non-atrophic gastritis	43	F	Pasto
CR017	Non-atrophic gastritis	45	M	Pasto
CR023	Non-atrophic gastritis	42	F	Pasto
CR032	Non-atrophic gastritis	24	F	Sandona

**Table 2:** The results of alpha diversity analyses (ACE, Simpson, and Shannon) according to different variables. The type of statistical test applied, the corresponding statistic values, and *p*-values are shown. Differences with *p* < 0.05 were considered significant.

Variable	Index	Test	Estimate	*p*-Value
**Group**	ACE	Wilcoxon rank sum exact test	W = 47	0.7616
	Simpson		W = 59	0.5267
	Shannon		W = 54	0.3739
***Helicobacter***	ACE	Student’s *t*-test	*t* = −0.0090795	0.9928
			df = 27.186	
	Simpson	Wilcoxon rank sum exact test	W = 150	**0.0276**
	Shannon		W = 159	**0.008319**

**Table 3:** Results of permutational analysis of variance (PERMANOVA) applied to beta diversity using Bray–Curtis distance. R^2^, F-statistic, and *p*-values are shown for each evaluated variable.

Variable	R^2^	*p*-Value	F-Statistic
** Group**	0.5825	0.104	1.7318
***Helicobacter***	0.10713	0.012 *	3.3597

Conventions: *p* < 0.05, *; *p* > 0.05, not significant.

## Data Availability

The following supporting information can be downloaded at NCBI (BIOPROJECT: PRJNA1482574): https://www.ncbi.nlm.nih.gov/bioproject/PRJNA1482574/ (accessed on 26 June 2026).
